# Objectively measured physical activity of USA adults by sex, age, and racial/ethnic groups: a cross-sectional study

**DOI:** 10.1186/1479-5868-6-31

**Published:** 2009-06-03

**Authors:** Marquis S Hawkins, Kristi L Storti, Caroline R Richardson, Wendy C King, Scott J Strath, Robert G Holleman, Andrea M Kriska

**Affiliations:** 1 Department of Epidemiology, University of Pittsburgh, 130 Desoto Street, Pittsburgh, Pennsylvania 15261, USA; 2 Ann Arbor VA Medical Center, PO Box 130170, Ann Arbor, Michigan, 48113-0170, USA; 3 Department of Family Medicine, University of Michigan, 1018 Fuller St, Ann Arbor, Michigan, 48104-1213, USA; 4 Department of Human Movement Sciences, University of Wisconsin, Enderis Hall, Room 435 PO Box 413, Milwaukee, Milwaukee, Wisconsin 53201-0413, USA

## Abstract

**Background:**

Accelerometers were incorporated in the 2003–2004 National Health and Nutritional Examination Survey (NHANES) study cycle for objective assessment of physical activity. This is the first time that objective physical activity data are available on a nationally representative sample of U.S. residents. The use of accelerometers allows researchers to measure total physical activity, including light intensity and unstructured activities, which may be a better predictor of health outcomes than structured activity alone. The aim of this study was to examine objectively determined physical activity levels by sex, age and racial/ethnic groups in a national sample of U.S. adults.

**Methods:**

Data were obtained from the 2003–2004 NHANES, a cross-sectional study of a complex, multistage probability sample of the U.S. population. Physical activity was assessed with the Actigraph AM-7164 accelerometer for seven days following an examination. 2,688 U.S. adults with valid accelerometer data (i.e. at least four days with at least 10 hours of wear-time) were included in the analysis. Mean daily total physical activity counts, as well as counts accumulated in minutes of light, and moderate-vigorous intensity physical activity are presented by sex across age and racial/ethnic groups. Generalized linear modeling using the log link function was performed to compare physical activity in sex and racial/ethnic groups adjusting for age.

**Results:**

Physical activity decreases with age for both men and women across all racial/ethnic groups with men being more active than women, with the exception of Hispanic women. Hispanic women are more active at middle age (40–59 years) compared to younger or older age and not significantly less active than men in middle or older age groups (i.e. age 40–59 or age 60 and older). Hispanic men accumulate more total and light intensity physical activity counts than their white and black counterparts for all age groups.

**Conclusion:**

Physical activity levels measured objectively by accelerometer demonstrated that Hispanic men are, in general, more active than their white and black counterparts. This appears to be in contrast to self-reported physical activity previously reported in the literature and identifies the need to use objective measures in situations where the contribution of light intensity and/or unstructured physical activity cannot be assumed homogenous across the populations of interest.

## Background

Physical activity can prevent and delay the onset of chronic diseases, such as diabetes and heart disease [1,2]. Despite this, inactivity is common in the United States. National surveillance systems have included measures of physical activity allowing researchers to measure trends in physical activity as well as identify groups that are physically inactive. Historically, national reports, such as the Behavior Risk Factor Surveillance System (BRFSS) and National Health and Nutrition Examination Survey (NHANES), have incorporated subjective measures (e.g. questionnaires) because of their low cost and ease of use, which makes them ideal for large population studies. However, objective measures, such as pedometers, which count steps, and accelerometers, which measure movement intensity, are starting to be utilized to assess physical activity levels in population studies [3-6].

Both subjective and objective assessment tools have advantages and disadvantages depending on the population being studied and the research question being investigated [7]. Subjective measures often provide detailed information regarding type of activity. However, because they rely on self-report of activity they are subject to response bias and recall bias. In particular, they have limited accuracy at capturing activities that are unstructured, and of light intensity [8], both of which tend to be performed in greater frequency in women and in older populations [9-11]. Thus, using a subjective measure of physical activity may not provide an accurate description of activity in these groups. It is important to measure total physical activity (which includes light intensity and unstructured activities) because it may have a greater impact on several health outcomes, such as diabetes, compared to moderate-to-vigorous physical activity alone [12-14]. Objective measures, such as accelerometers, do not provide detailed information on the type of activities being performed. However, they do record physical activity across all intensities and are not subject to the biases of self-report. In addition, accelerometers can be used to estimate the time spent in light, moderate, and high intensity physical activity and the contribution of each to total physical activity [15].

Accelerometers were incorporated in the 2003–2004 NHANES study cycle for objective assessment of physical activity. This is the first time that objective physical activity data are available on a nationally representative sample of U.S. residents. A previous effort by Troiano et al focuses on physical activity at moderate-to-vigorous levels of intensity describing, on a population level, who's meeting the Surgeon General's recommendations of physical activity using this accelerometry data from NHANES [6]. In contrast, given the potential importance of total physical activity, the current effort includes assessments of light intensity activity and moderate-vigorous intensity activity to examine differences in physical activity by sex, age and racial/ethnic groups in a national sample of adults.

## Methods

### Study Population and Procedures

NHANES is a cross-sectional observational study conducted by the National Center for Health Statistics of the Centers for Disease Control that uses a stratified, multistage probability design to obtain a nationally representative sample of the U.S. household population [16]. The survey population includes randomly selected neighborhoods in the United States. From these neighborhoods, random households are selected to incorporate individuals in this study. During the 2003–2004 NHANES study cycle, 12,761 individuals were selected for recruitment. Of those selected, 10,122 individuals (79%) agreed to participate in the study.

**Figure 1:**
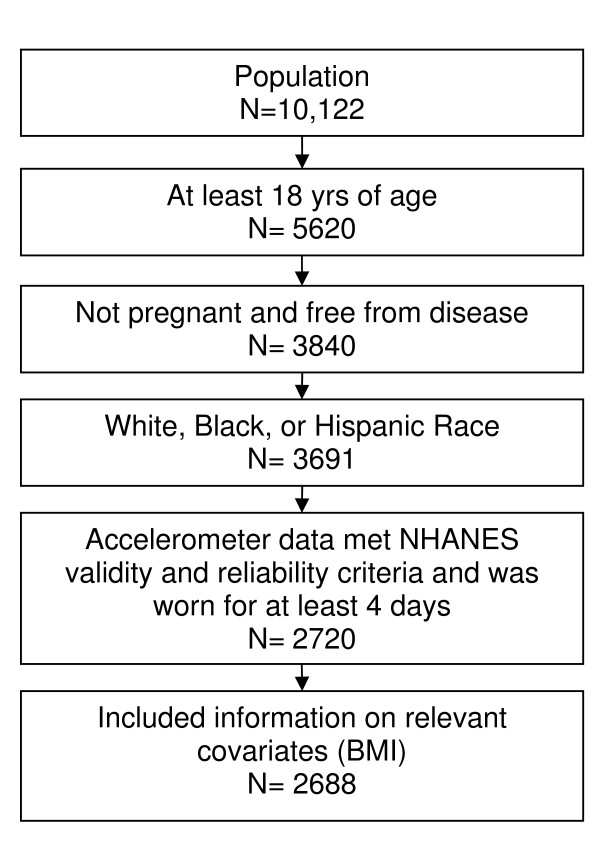
**Study inclusion flow chart**.

NHANES 2003–2004 consisted of an interview, physical examination, and laboratory tests conducted in mobile examination centers (MEC) by trained staff. Physical activity assessment was restricted to NHANES participants who were not prevented by impairments from walking or wearing an accelerometer. Our analysis was further restricted to individuals who were at least 18 years of age, not pregnant, and never reported having had a stroke, congestive heart failure, angina, emphysema, chronic bronchitis, or renal dialysis to exclude individuals whose physical activity was limited because of illness or health status (N = 3840). Fewer than 5% of the remaining participants reported a race other than non-Hispanic white, non-Hispanic black or Hispanic so they were subsequently excluded from this analysis due to insufficient sample size (N = 149). Participants who had at least four days of valid accelerometer data with relevant covariates were used in the analysis (N = 2688) (Figure 1).

### Measures

#### Physical Activity

Physical activity was assessed with the Actigraph AM-7164 accelerometer (formerly the CSA/MTI AM-7164, manufactured by ActiGraph of Ft. Walton Beach, Florida, USA), which is a pager size device powered by a small lithium battery. The accelerometers were attached to an elasticized belt and worn on the right hip. The accelerometer measures the duration and intensity of physical activity by capturing the magnitude of acceleration (intensity) and summing up the magnitudes (intensity counts) within a specified time interval (epoch). A one-minute epoch was used by NHANES.

Participants were asked to wear the device for seven days while they were awake, and to take it off for swimming or bathing. Monitors were returned by express mail to NHANES, where data were downloaded from the device, and the device was checked to determine whether it was still within the manufacturer's calibration specifications using an Actigraph calibrator.

NHANES used standardized data quality procedures to assess the validity and reliability of the accelerometry data, which are described in more detail elsewhere [17]. In brief, participants with at least four days in which the accelerometer was worn for at least 600 minutes are included in analysis. Any block of time greater than or equal to 60 minutes where the activity count was equal to zero was considered time when the monitor was not worn. Based on a previous calibration study by Freedson and colleagues, a minute of accelerometer data was coded as sedentary if it contained less than 260 activity counts. A minute was coded as light physical activity if it recorded an accelerometer count between 260–1951. A minute was coded as moderate-vigorous intensity if activity counts were greater than or equal to 1952 [18]. Counts derived during minutes of light and moderate-vigorous activity respectively, were individually summed and divided by the number of days worn to calculate daily averages for those specific intensities. Total physical activity was the sum of the counts derived during minutes of light and moderate-vigorous activity.

### Demographics and Anthropometrics

Participants were categorized as non-Hispanic white, non-Hispanic black, and Hispanic (including Mexican American and other Hispanic) based on self-report. Age in years was calculated from self-report. Participants over the age of 85 were assigned the age of 85 to protect confidentiality. For descriptive purposes, activity was tabulated by age groups: 18–39, 40–59 and 60 or more years. Body Mass Index (BMI) was calculated (BMI) (kg/m^2^) from height and weight, which were measured at MEC using standardized protocol and equipment. General health status was self-reported as excellent, very good, good, fair, or poor.

#### Data Analyses

Statistical analyses were performed using STATA 10.0 (StataCorp Stata Statistical Software: Release 10.1. StataCorp LP: College Station, TX, 2007). For all analyses, significance was set at an alpha level of <0.05. Unadjusted values were calculated stratified by sex, race/ethnicity and age groups. Generalized linear modeling using the log link function was performed to compare total, light, and moderate-vigorous activity counts by sex and race/ethnic groups adjusting for age and age-squared because it's non-linear relationship with physical activity. To determine whether there were interactions between age and race in either males or females interaction terms were added to the models. All analysis took into account the complex survey design of NHANES, which includes weighting, stratification and clustering.

## Results

Demographic characteristics of the 2,688 participants are presented in Table 1. Participants were stratified by sex and race/ethnicity to form six groups that consisted of white men, black men, Hispanic men and white women, black women and Hispanic women. As a group, Hispanic men and women on average appeared to be younger than their white and black counterparts. The majority of the participants in the study were in good or very good health which is reflective of inclusion criteria because we whose physical activity may possibly be limited by underlying conditions.

**Table 1:** Descriptive characteristics of the study population stratified by sex

	**Male**			**Female**		
	White (N= 714)	Black (N= 277)	Hispanic (N= 369)	White (N= 713)	Black (N= 258)	Hispanic (N= 357)
General characteristics						
BMI (kg/m^2^)	27.9 ± 3.4	28.4 ± 8.0	27.6 ± 5.3	27.4 ± 4.4	31.5 ± 8.4	28.1 ± 7.4
						
Age (years)	45.5 ± 10.9	42.1 ± 19.7	38.8 ± 16.0	47.7 ± 11.3	44.2 ± 17.3	40.1 ± 18.5
Health Status (%)						
						
Excellent	14.7	15.2	15.3	7.9	15.7	11.3
Very Good	23.0	41.4	31.2	25.5	41.3	31.3
Good	41.8	33.3	40.1	38.4	32.8	32.9
Fair	19.6	8.7	11.1	25.4	8.1	23.4
poor	1.1	1.4	2.3	2.7	2.1	1.1
						
Wear time (mean min/day)	1000 ± 115	1051 ± 240	996 ± 183	973 ± 80	1025 ± 228	981 ± 161

All values are means + standard deviations unless labeled otherwise

Age-adjusted physical activity counts by race/ethnicity and gender are presented in Table 2. Hispanic men appeared to have both higher age-adjusted total and light activity counts compared to white and black men (p < 0.01). Moderate-vigorous physical activity followed a similar pattern of higher counts in Hispanic men but the difference was not statistically significant (p = 0.13). Likewise, Hispanic women appeared to have significantly higher counts of light intensity physical activity compared to their white and black counterparts (p = 0.02). Total activity followed a similar pattern of higher counts in Hispanic women but the difference did not reach statistical significance (p = 0.14). Moderate -vigorous physical activity counts appeared to be similar in women (p = 0.50). There was a statistically significant interaction with regard to age for Hispanic men, which suggests that Hispanic men experience a faster decline in moderate-vigorous physical activity with increasing age (p = 0.03). No other significant interactions were noted (data not shown).

**Table 2:** Daily age-adjusted activity counts (thousands) by intensity level and by sex and race

	**Hispanic**	**White**	**Black**	**p-value**
MEN				
Total physical activity	303.9 (288.1, 320.6)	258.0 (243.6, 273.3)	266.4 (239.2,296.6)	0.001
Light Physical Activity	195.3 (185.9, 205.2)	165.4 (156.5, 174.9)	168.3 (153.9, 184.2)	0.001
Moderate-Vigorous Physical Activity	106.6 (93.0, 122.3)	90.0 (81.1, 100.0)	96.2 (78.1, 118.7)	0.12
WOMEN				
Total physical activity	217.9 (198.4, 239.3)	206.2 (196.0, 216.9)	192.6 (177.1, 209.4)	0.14
Light Physical Activity	159.3 (151.9, 167.2)	149.0 (142.6, 155.8)	142.6 (133.1, 152.9)	0.02
Moderate-Vigorous Physical Activity	55.7 (44.9, 69.1)	55.1 (48.8, 62.3)	48.3 (38.6, 60.6)	0.49

All values are means (95% confidence intervals)

Physical activity counts per day for total, light, and moderate-to-vigorous intensity stratified by age, sex, and race/ethnicity are presented in Table 3. When stratifying by age group, the pattern of higher activity counts in Hispanic men remained, however, significance was only reached in total and light activity in the youngest age groups (p = 0.001 and p = 0.002 respectively). There was also a pattern for higher activity counts in Hispanic women in the middle age groups, reaching significance for total and light activity (p = 0.006 and p = 0.003 respectively). Women appeared to have similar activity counts across race/ethnicity groups in the younger and older age groups.

**Table 3:** Daily physical activity counts (thousands) by intensity level and by sex, race and age groups

		**Male**			**Female**		
Physical Activity Level	Age	Hispanics	Whites	Blacks	Hispanics	Whites	Blacks
Total physical activity	18–39	**411.7** **(385.6, 437.8)**	**337.9** **(318.4, 357.5)**	**338.7** **(291.4, 386.0)**	249.6 (228.0, 271.2)	263.5 (247.2, 279.7)	234.4 (208.0, 260.7)
	40–59	319.2 (288.9, 349.6)	293.0 (268.8, 317.2)	317.0 (270.5, 363.6)	**286.7** **(253.5, 319.8)**	**222.2** **(209.4, 234.9)**	**224.8** **(198.3, 251.2)**
	60+	211.6 (169.3, 254.0)	182.4 (162.5, 202.3)	171.3 (148.2, 194.3)	155.7 (134.0, 177.4)	158.8 (147.2, 170.4)	145.4 (130.7, 160.2)
Light Physical Activity	18–39	**239.8** **(224.9, 254.8)**	**197.6** **(178.7, 216.5)**	**189.8** **(168.1, 211.5)**	170.4 (158.5, 182.3)	173.5 (163.3, 183.7)	160.3 (148.7, 171.9)
	40–59	210.3 (183.1, 237.4)	187.5 (176.0, 199.0)	199.0 (176.7, 221.4)	**196.3** **(185.2, 207.4)**	**162.5** **(155.4, 169.7)**	**163.3** **(143.6, 183.1)**
	60+	149.4 (124.6, 174.2)	131.8 (119.9, 143.8)	134.6 (116.1, 153.0)	129.7 (113.4, 146.0)	128.4 (118.0, 138.7)	124.5 (112.1, 136.9)
Moderate-Vigorous Physical Activity	18–39	171.9 (147.6, 196.1)	140.3 (123.4, 157.2)	148.9 (114.2, 183.5)	79.2 (63.2, 95.2)	90.0 (73.4, 106.6)	74.1 (50.2, 98.0)
	40–59	109.0 (95.0, 123.0)	105.5 (88.9, 122.1)	117.9 (84.0, 152.0)	90.4 (62.5, 118.2)	59.7 (49.3, 70.0)	61.4 (44.3, 78.6)
	60+	62.2 (40.0, 84.4)	50.6 (40.7, 60.4)	36.7 (28.8, 44.7)	26.0 (18.6, 33.4)	30.5 (25.5, 35.4)	21.0 (13.2, 28.7)

Significant differences in activity counts by gender are indicated with bold print

All values are means and 95% confidence interval

## Discussion

The current study examined objectively measured physical activity data collected on a nationally representative sample of U.S. adults and thus provided a unique opportunity to investigate total physical activity and various levels of physical activity intensity across racial/ethnic and sex groups. The most striking findings were the relatively higher amounts of physical activity among Hispanic men and women compared to their male and female counterparts.

This current effort indentified a pattern of higher physical activity levels in Hispanic men compared to white and black men. The results also indicate that Hispanic women engage in more light physical activity compared to their white and black counterparts. This is in contrast to previous results from national surveys, which have indicated that Hispanic men and women are less active compared to their white and black counterparts. Results from the 1994–2004 BRFSS showed that Hispanic men and women reported greater amounts of inactivity than white men and women [19]. However, the pattern of higher objectively measured physical activity in Hispanic men was also shown in a study by Bennett et al which showed higher pedometer step counts for Hispanic men compared to their white and black counterparts, though not reaching significance [20]. A previous paper by Troiano et al using NHANES accelerometry data also showed that Hispanic men and women were more active than white and black men and women at moderate-to-vigorous levels of intensity [6]. This effort expands on the work by Troiano by including total activity encompassing all levels of intensity and comes to the same conclusion. Another effort using the NHANES population was conducted by Matthews et al which looked at time spent in sedentary activity using the accelerometer [21]. The results of the study indicated that Hispanic men and women spent less time in sedentary activity than their white and black counterparts.

Additionally, physical activity was found to be inversely associated with age at all levels of intensity in all racial/ethnic groups with the exception of Hispanic women. This finding is consistent with results of previous studies using subjective data [22,23] suggesting a general pattern of decreasing physical activity levels with increasing age. This finding is also in line with the National Health Interview Survey (NHIS) which showed that adults in the 65+ years age group were about five times more likely than adults in the 18–24 years age group to report never being physical active [24]. Likewise, results from the Behavioral Risk Factor Surveillance System (BRFSS) indicated that 35% of men and 54% of women aged 75+ years reported engaging in no physical activity compared to 15% of men and 20% women aged 18–29 years who reported engaging in no physical activity [19]. A national representative study of Swedish adults by Hagstromer et al, using accelerometery, also noted that physical activity levels decreased with age [4].

The 2003–2004 NHANES accelerometer effort found men to be more physically active than women across all age groups with the exception of Hispanic women in the middle age group. There were no significant differences in total, light, or moderate-vigorous activity when comparing men to Hispanic women (p = 0.36, p = 0.34, and p = 0.61 respectively) in the middle age group. These findings need further investigation into what factors cause higher levels of activity in Hispanic women in the middle age group.

There are important limitations worth noting when interpreting accelerometry data. Accelerometers do not provide qualitative information on what types of physical activities are being performed (household, transportation, leisure, etc.). Future studies will be needed to identify the specific areas in which Hispanic male and females accumulate more activity than their white and black counterparts. Accelerometers are also unable to capture upper arm movement. Thus, a portion of activity can be missed (e.g. weight lifting). Walking gait must also be taken into consideration because it can affect the validity of accelerometer counts, especially in older adults. For example, some accelerometers are more likely to undercount activity in adults with a non-standard gait [25]. This bias should be minimal because we excluded individuals whose physical activity would likely be affected by illness or health status.

## Conclusion

The current study is one of only a few studies utilizing an objective assessment of physical activity in a large representative sample of U.S. adults. Findings from this study will serve as a basis for comparison of objectively measured physical activity across sex and racial/ethnic groups in future efforts. It is important to be able to identify pockets of the population that are currently inactive to be able to focus future intervention efforts. The reasons that certain groups may be inactive may be are likely multi-factorial and may be related to many factors including socio-economic factors and the built environment which are not identified in this study. Furthermore, this study identifies the need to use objective measures of physical activity in situations where the contribution of light intensity and/or unstructured physical activity cannot be assumed homogenous within the population of interest.

## Competing interests

The authors declare that they have no competing interests.

## Authors' contributions

MH contributed to the design of the manuscript as well as the analysis and interpretation of data. MSH also contributed to the revisions of the manuscript content and gave approval of the final version to be published.

KS contributed to the design of the manuscript as well as the analysis and interpretation of data. KLS also contributed to the revisions of the manuscript content and gave approval of the final version to be published.

CR contributed to the design of manuscript as well as the interpretation of the data. CRR also contributed to the revision of manuscript content and gave approval of the final version to be published.

WK contributed to the design of the manuscript as well as the analysis and interpretation of data. WCK also contributed to the revision of the manuscript content and gave approval of the final version to be published.

SS contributed to design of the manuscript as well as the interpretation of the data and was also involved in the revising manuscript content. SJS also gave approval for the final version to be published.

RH contributed to the design of the manuscript as well as the acquisition and analysis of data. RGH also contributed to the revision of manuscript content and gave final approval of the version to be published.

AK contributed to the design of the manuscript as well as the analysis and interpretation of data. AMK also contributed to the revision of the manuscript content and gave approval of the final version to be published.

## References

[B1] Blair SN (1993). C.H. McCloy Research Lecture: physical activity, physical fitness, and health. Res Q Exerc Sport.

[B2] Pate RR (1995). Physical activity and public health. A recommendation from the Centers for Disease Control and Prevention and the American College of Sports Medicine. Jama.

[B3] De Cocker K, Cardon G, De Bourdeaudhuij I (2007). Pedometer-determined physical activity and its comparison with the International Physical Activity Questionnaire in a sample of Belgian adults. Res Q Exerc Sport.

[B4] Hagstromer M, Oja P, Sjostrom M (2007). Physical activity and inactivity in an adult population assessed by accelerometry. Med Sci Sports Exerc.

[B5] Troiano RP (2007). Large-scale applications of accelerometers: new frontiers and new questions. Med Sci Sports Exerc.

[B6] Troiano RP (2008). Physical activity in the United States measured by accelerometer. Med Sci Sports Exerc.

[B7] Kriska A, Caspersen CJ (1997). Introduction to a Collection of Physical Activity Questionnaires. Med Sci Sports Exerc.

[B8] Leenders NY (2001). Evaluation of methods to assess physical activity in free-living conditions. Med Sci Sports Exerc.

[B9] Ainsworth BE (2000). Evaluation of the kaiserphysical activity survey in women. Med Sci Sports Exerc.

[B10] King AC (2001). Interventions to promote physical activity by older adults. J Gerontol A Biol Sci Med Sci.

[B11] King AC, Rejeski WJ, Buchner DM (1998). Physical activity interventions targeting older adults. A critical review and recommendations. Am J Prev Med.

[B12] Ham SA (2007). Physical activity patterns among Latinos in the United States: putting the pieces together. Prev Chronic Dis.

[B13] Healy GN (2007). Objectively measured light-intensity physical activity is independently associated with 2-h plasma glucose. Diabetes Care.

[B14] Sesso HD (2007). Invited commentary: a challenge for physical activity epidemiology. Am J Epidemiol.

[B15] Mathie MJ (2004). Accelerometry: providing an integrated, practical method for long-term, ambulatory monitoring of human movement. Physiol Meas.

[B16] Centers for Disease Control and Prevention, N.C.f.H.S (2003). NHANES 2003–2004 Public Data General Release File Documentation. http://www.cdc.gov/nchs/data/nhanes/nhanes_03_04/general_data_release_doc_03-04.pdf.

[B17] Centers for Disease Control and Prevention, N.C.f.H.S (2004). Laboratory Procedures Manual.

[B18] Freedson PS, Melanson E, Sirard J (1998). Calibration of the Computer Science and Applications, Inc. accelerometer. Med Sci Sports Exerc.

[B19] (2005). Trends in leisure-time physical inactivity by age, sex, and race/ethnicity – United States, 1994–2004. MMWR Morb Mortal Wkly Rep.

[B20] Bennett GG (2006). Pedometer-determined physical activity among multiethnic low-income housing residents. Med Sci Sports Exerc.

[B21] Matthews CE (2006). Amount of time spent in sedentary behaviors in the United States, 2003–2004. Am J Epidemiol.

[B22] Jones DA (1998). Moderate leisure-time physical activity: who is meeting the public health recommendations? A national cross-sectional study. Arch Fam Med.

[B23] Marshall SJ (2007). Race/ethnicity, social class, and leisure-time physical inactivity. Med Sci Sports Exerc.

[B24] Barnes PM, Schoenborn CA (2003). Physical activity among adults: United States, 2000.

[B25] Storti KL (2008). Gait speed and step-count monitor accuracy in community-dwelling older adults. Med Sci Sports Exerc.

